# Interventions to mitigate indoor air pollution: A cost-benefit analysis

**DOI:** 10.1371/journal.pone.0257543

**Published:** 2021-09-24

**Authors:** Muhammad Irfan, Michael P. Cameron, Gazi Hassan

**Affiliations:** 1 School of Economics and Management, Xiamen University Malaysia, Sepang, Malaysia; 2 School of Accounting, Finance, and Economics, University of Waikato, Hamilton, New Zealand; Universita degli Studi del Molise, ITALY

## Abstract

Globally, around three billion people depend upon solid fuels such as firewood, dry animal dung, crop residues, or coal, and use traditional stoves for cooking and heating purposes. This solid fuel combustion causes indoor air pollution (IAP) and severely impairs health and the environment, especially in developing countries like Pakistan. A number of alternative household energy strategies can be adopted to mitigate IAP, such as using liquefied petroleum gas (LPG), natural gas, biogas, electric stoves, or improved cook stoves (ICS). In this study, we estimate the benefit-cost ratios and net present value of these interventions over a ten-year period in Pakistan. Annual costs include both fixed and operating costs, whereas benefits cover health, productivity gains, time savings, and fuel savings. We find that LPG has the highest benefit-cost ratio, followed by natural gas, while ICS has the lowest benefit-cost ratio. Electric stoves and biogas have moderate benefit-cost ratios that nevertheless exceed one. To maximize the return on cleaner burning technology, the government of Pakistan should consider encouraging the adoption of LPG, piped natural gas, and electric stoves as means to reduce IAP and adopt clean technologies.

## Introduction

Currently, almost three billion people in low- and middle-income countries do not have access to clean or modern energy sources and hence depend upon solid fuels such as firewood, biomass, crop residues, coal, and charcoal for cooking and heating [1]. When these solid fuels burn, they emit a multitude of complex chemicals including formaldehyde, nitrogen dioxide, carbon monoxide, cilia toxic, polycyclic aromatic hydrocarbons, and other inhalable particulates [2, 3]. These pollutants lead to adverse effects on health and the environment. Alarmingly, the overall household consumption of solid fuels is expected to continue increasing until 2030 [4].

When conducted indoors, biomass combustion causes indoor air pollution (IAP) and due to this almost four million people die prematurely each year [5] and millions more face serious diseases such as lung infections, asthma, tuberculosis, sinus problems, cardiovascular disease, and cancer [6–8]. In total, pollution (of which IAP is one example) is chiefly responsible for more deaths than AIDS, tuberculosis, obesity, malaria, child and maternal malnutrition, alcohol, road accidents, or war [1]. Moreover, various studies have found a positive association between IAP and ill health, and have suggested that use of cleaner fuels can save lives, and lead to health and environmental benefits [9–13].

IAP arises from a combination of the choice of fuel by a household, the way that the household uses the fuel, and the characteristics of the living environment. Thus, interventions to reduce IAP can be categorized into three types: (1) Interventions affecting the source of pollution; (2) Interventions affecting the living environment; and (3) Interventions that change users’ behavior [14, 15]. In the first type of intervention, IAP can be reduced by switching from solid fuel to cleaner fuels. For instance, households may switch from using coal, firewood, animal dung, or crop residues to electricity, liquefied petroleum gas (LPG), piped natural gas, or biogas. An example of the second type of intervention is improving ventilation of the cooking and living area. Examples of the third type of intervention include drying firewood before use, keeping young children away from smoke, and blowing out the fire immediately after cooking.

Although, Malla *et al*. (2011) [16] and Hutton *et al*. (2007) [17] provide cost-benefit evaluations at the regional (multi-country) level, every country has different local behaviours in terms of energy consumption, as well as differences in infrastructure and climate, which can impact the costs and benefits of interventions [13]. Moreover, the consumption of solid fuels differs across low- and middle-income countries (LMICs) (and is much higher than in developed countries), and is often higher in rural areas than in urban areas [18]. Hence, the negative effects arising from IAP are not distributed evenly across the population [1]. Moreover, despite the availability of potentially cost-effective interventions, these have yet to be adopted in many developing countries like Pakistan. Part of the reason for this may be a lack of understanding of the costs and benefits of these interventions in local conditions. We aim to contribute to the adoption of effective interventions to mitigate IAP, by demonstrating the net benefits of these interventions.

Due to heterogeneity in the use of solid fuels and the impacts of IAP between LMICs, and between urban and rural areas of LMICs, investigating the effects of interventions to address IAP requires careful selection of exemplar case studies. In this paper, we use Pakistan as a case study for our analysis of the costs and benefits of adopting cleaner burning technologies. Pakistan provides a good exemplar for other developing countries, because it has diverse household energy options, and households currently employ a mix of clean and solid fuel energy sources [19]. Generally, in Pakistan, piped natural gas, LPG, firewood, crop residues, and animal dung are the main energy sources for cooking food, whereas electricity is rarely used for cooking. Additionally, urban households have better access to piped natural gas and electricity [19]. The proportion of urban households in Pakistan was around 36.9 percent and GDP per capita was 1,284 USD in 2019 [20]. Moreover, Pakistan has suitable microdata available for analysis, which are not available in all developing countries. The findings of this study may therefore also provide some guidance for other low and middle-income countries, especially those in South Asia.

Our study is one of the first to compare a range of possible IAP mitigating technologies at the country level, rather than at a more aggregated multi-country region. This allows us to better consider differences in adoption between rural and urban households. We evaluate five interventions, including four examples of the first type of intervention (universal adoption of LPG; natural gas; electric stove; or biogas), and one example of the second type of intervention (universal adoption of improved cook stoves (ICS)). Because of data unavailability, it is not possible to address third type of intervention (changes in user behaviour). Economic evaluation through cost-benefit analysis is a widely used analytical tool for comparing the benefits and costs of interventions [17]. We follow the guidelines of the World Health Organization to estimate the benefit-cost ratio (BCR) and net present value (NPV) of the five interventions, and find that universal adoption of LPG has the highest benefit-cost ratio of the five interventions we evaluate. However, of the five interventions, improved cook stoves are the only intervention where benefits do not exceed costs.

### Literature review

There is very limited literature on cost-benefit analysis of household energy interventions. Moreover, extant studies tend not to evaluate a wide range of the available choices a household can adopt to reduce IAP. However, Mehta and Shahpar (2004) [14] examined the results of two major interventions (providing access to cleaner fuels; and providing access to ICS) in six epidemiologic sub-regions (Africa Region, Region of the Americas, Eastern Mediterranean Region, European Region, South East Asian Region, Western Pacific Region). They focused on two main health outcomes associated with IAP: (1) acute lower respiratory infections in young children under five years of age; and (2) chronic obstructive pulmonary disease in adults aged over twenty years. They estimated the cost using a costing template developed by WHO, and found that these interventions could reduce the burden of diseases associated with IAP and save 500–600 international dollars per year per household (international dollars have the same purchasing power as a US dollar). They concluded that providing access to cleaner fuels has a greater positive health effect than ICS, although there were also significant health benefits arising from ICS adoption.

Hutton, Rehfuess and Tediosi (2007) [17] also applied cost-benefit analysis, for the same epidemiologic sub-regions as Mehta and Shahpar (2004) [14], to evaluate two interventions: (1) access to cleaner fuels; and (2) more efficient stoves. They also followed the WHO’s guidelines for the estimation of economic costs and benefits. Costs included fuel costs, stove costs, program costs, and operational costs, while benefits included reduced health related expenditures, productivity gains, time savings, and environmental benefits. A sensitivity analysis was also carried out to explicitly estimate the uncertainty in the results. Surprisingly, for the Eastern Mediterranean Region-D (D indicates high adult and child mortality in the region and EMR-D, the region that includes Pakistan) the BCR for LPG in urban areas was less than 1, while for ICS in both urban and rural areas the BCR was less than 1. This implies that the LPG and ICS interventions are not beneficial for the EMR-D region on average. In other words, in this region the net cost of the interventions is higher than the net benefits. On the other hand, Jeuland and Pattanayak (2012) [21] carried out an extensive review of literature on cost-benefit analysis for ICS and found that the net benefits for households were mostly positive for ICS; however, sometimes they can be negative when health benefits are low because of poorly performing stoves. Nevertheless, the net benefits for the households can be higher when they reap indirect health benefits because of avoiding infectious diseases. For instance, due to the use of ICS the incidence of acute respiratory infection may decrease at the community level. Unfortunately, there is a severe lack of scientific studies that demonstrate the indirect health benefits arising from the use of ICS. Likewise in Orissa (India), Hanna et al. (2016) [22] conducted a large scale experimental study using a randomized trial to explore the impact of ICS usage on health and environment. They found that the inhalation of smoke falls in the first year after adoption, however, by year two and thereafter there were no health and environmental benefits recorded.

Some studies such as Zhao et al., (2018) [23] and Liu et al., (2021) [24] have suggested the use of air purifiers to control IAP in China. However, this may not be a useful option to reduce IAP in the case of Pakistan. First, households may be reluctant to buy an air purifier (even if the health benefits outweigh the costs [24]) due to its high initial cost. Most households that use solid fuel are low income, hence even a low initial cost can be a significant barrier to adoption. Second, air purifiers require electricity to operate, and so the operating cost may be high in Pakistan because of the high per unit cost of electricity. Third, the supply of electricity is not consistent because of blackouts and roughly 10% of households in Pakistan do not have an electricity connection [19] and therefore, the use of an air purifier will not be possible for those households. Above all, it is likely to be far better to use cleaner fuels to reduce IAP than using dirty fuels and then using an air purifier to reduce the resulting IAP.

There are only a handful studies that have evaluated the costs and benefits of interventions at the country level, such as Malla et al (2011) [16] in Kenya, Sudan, and Nepal, Abbas et al (2017) [25] in Pakistan, Aunan et al (2013) [26] in China, Isihak, Akpan and Adeleye (2012) [27] in Nigeria, García-Frapolli et al (2010) [28] in Mexico, and Rivoal and Haselip (2017) [29] in Tanzania. Table 1 briefly summarises the results. In all of these studies, the BCRs exceed one, demonstrating that all the interventions are net beneficial. The measured BCRs range from 1.55 (LPG in Tanzania) to 21.4 (a combination of ICS, smoke hoods, and LPG in Kenya). However, none of these studies have evaluated more than three interventions, and most evaluated just a single intervention. Consequently, some useful interventions such as natural piped gas and electric stoves remain relatively unexplored, while others lack a standardized within-country comparison. Our study fills this significant gap by providing analysis for five IAP mitigating technologies, including piped natural gas and electric stoves, which have thus far been largely ignored in the literature. This is important because both piped natural gas and electric stoves are important alternatives for governments to consider.

**Table 1:** Summary of estimated benefit-cost ratios for IAP interventions in previous studies.

Study	Country	Interventions	Results
Malla et al (2011) [16]	Kenya	Combination of ICS, smoke hoods and LPG	Kenya: BCR = 21.4, NPV = 977 USD
			Sudan: BCR = 2.5, NPV = 226.7 USD
	Sudan		
			Nepal: BCR = 1.4, NPV = 29.6 USD
	Nepal		
Abbas et al. (2017) [25]	Pakistan	Biogas	BCR = 1.55 to 2.04 for 10m^3^
Aunan et al. (2013) [26]	China	ICS	BCR = 3.3 to 14.7
Isihak et al. (2012) [27]	Nigeria	ICS	ICS: BCR = 2.57
		LPG	LPG: BCR = 2.70
García-Frapolli et al. 2010 [28]	Mexico	ICS	BCR = 9 to 11.4 (estimated for 7 and 14 years)
Rivoal and Haselip (2017) [29]	Tanzania	LPG	BCR = 1.69 to 1.76 (over 10 years)
			BCR = 1.55 to 1.6 (over 5 years)
Limmeechokchai and Chawana 2007 [30]	Thailand	Biogas	BCR = 1.58 to 1.67
			NPV = 852 to 5271 USD at 12%

Notes: BCR = Benefit-Cost Ratio; NPV = Net Present Value.

## Materials and methods

### Data

Estimating the benefit-cost ratio for IAP interventions requires estimates of both benefits and costs. We draw from various data sources to make those estimates. The Pakistan Social Living Measurement Survey (PSLM, 2014) was used to estimate the total consumption of solid fuels, prices of the fuels, and households’ dependence on clean and solid fuels [19]. To collect information about the costs and benefits of the interventions, we used data from the Rural Support Program Network (RSPN), Bio Energy Technology Application Pakistan (BETAPak), and Pakistan Council of Renewable Energy Technologies (PCRET). Mortality and morbidity data were obtained from the World Health Organization.

Demographic variables such as region, age, and working age were obtained from Pakistan Bureau of Statistics. The total population of children was obtained from the United Nation’s Population Division. Per-capita income, total population, and average household size were obtained from the World Bank [20]. Electricity prices and natural gas connection charges were obtained from the Ministry of Water and Supply, and Sui Northern Gas Pipelines Limited (SNGPL), respectively. Finally, the number of days spent in bed because of illness, fuel collection time, time spent on economic activity, the operating cost of biogas plants, LPG, natural gas, electricity, the fixed costs of LPG, natural gas, and electric stoves, and environmental related variables were constructed with the help of published research studies. Further details can be found in in S1 Table.

### Methods

For monetizing the cost and benefits, this study follows the guidelines of the World Health Organization [31]. All benefits and costs are presented on an annual basis in millions of rupees (Pakistani currency) and US dollars for the year 2014 (for the sake of simplicity and data availability), and benefit-cost ratios (BCRs) were calculated by dividing net discounted benefits by net discounted costs at the single household level. This analysis assumes 2014 as the first year of the intervention and forecasted an intervention period of 10 years through to the end of 2024. The choice of starting year of 2014 was because the household microdata that was used to construct various variables was from 2014. However, we don’t anticipate that large shifts in the BCRs would result from choosing an alternative starting year. To get the BCRs we divided ten years of discounted benefits by ten years of discounted costs using Eq (1):

BCR=∑t=1nBn1+in∑t=1nCn1+in (1)

where *B*_*n*_ is the total benefits in year *n*, *C*_*n*_ is the total costs in year *n*, *i* is the discount rate, and *n* is the number of years. In addition to BCRs, we estimated the NPV using Eq (2):

$$NPV=\sum_{t=1}^{n}\frac{NB_{n}}{{\left(1+i\right)}^{n}}-C_{0}$$ (2)

where NB_n_ is the net benefit in year *n* (so NB_n_ = B_n_−C_n_), and C_0_ is the initial investment cost.

All the benefits and costs occurring after 2014 were discounted to 2014 values using discount rates of 3%, 7.5%, and 12%. The use of three different discount rates allows us to test the sensitivity of the results to the choice of discount rate, 3% is a commonly applied ‘social discount rate’ in developed countries (e.g. EPA, 2000), but higher discount rates may be more appropriate in developing countries, e.g. 12% has previously been used in Pakistan [25]. Higher social discount rates necessarily reduce the present value of net benefits greater than the present value of net costs, which tend to occur closer to the present time. Thus, the BCRs estimated using higher social discount rates will be more conservative.

As noted earlier, five interventions were included in this study, across two main intervention types: (1) changing from solid fuel use to cleaner fuels (biogas; LPG; or piped natural gas); and (2) changing stove types (to ICS; or electric stoves). In first type of intervention, the same *type* of stove is suitable for each option (biogas, LPG, or piped natural gas), because the stoves use methane as a fuel source. However, electric stoves require electricity for functioning. They also do not emit harmful gases or create meaningful IAP. Adopting an ICS reduces but does not eliminate the use of solid fuels, but reduces IAP because of the resultant use of taller chimneys or better ventilation.

We assumed that there are two types of households: (1) those who currently use clean fuels for cooking and heating purposes; and (2) others who do not. This simplification means that we only need to consider the costs and benefits of adoption for those households that would actually be affected by the interventions. That is, the costs and benefits were evaluated only for those households that depended on solid fuels at the start of the period. Initially, we estimated the costs for an individual household and then extrapolated the cost to the whole population who depended upon solid fuels in 2014. About 55% of Pakistani households do not use piped natural gas or LPG, and most of those households are in rural areas (PSLM, 2014). Considering the total population in 2014 and taking an average of 6.7 household members per household [25], 15.22 million households out of 27.68 million households depended on solid fuels and used traditional or inefficient stoves for cooking and heating. We estimated the benefits by assuming *all* households that depend upon solid fuels (15.22 million households) adopted the intervention. Discounted costs and benefits were estimated as noted in the following two sections, for a period of ten years, except for ICS (ICS has three years of useable life).

Considerable uncertainty is anticipated in the results, because of the lack of generalizable data and the number of necessary assumptions employed in the model. We performed sensitivity analysis to tackle this uncertainty. Specifically, following Isihak et al. (2012) [27] we estimated the BCR and other measures for additional scenarios. In two optimistic scenarios, we increased total benefits (by 5 and 10 percent) from the base case benefits and reduced the total costs from the base case costs (by 5 and 10 percent). In two pessimistic scenarios, we reduced the total benefits (by 5 and 10 percent) from the base case and increased the costs (by 5 and 10 percent) compared to the base case costs.

### Costs

The costs of each intervention were estimated as one-off fixed costs (installation, stove costs, etc.) occurring at the start of the period, and annual operating costs. Specific details for each intervention are detailed below.

#### Costs of biogas digesters

This intervention requires the installation of a biogas digester, as well as a new methane stove. First, there are different sizes of biogas plants available in Pakistan, ranging from 4 to 25 cubic meters. The median and most commonly installed size of biogas plant is 10 m^3^, and this size of plant is sufficient to fulfil the energy demand for an average family (6–8 members) [25]. We used the cost for a fixed dome digester, rather than a floating drum or flexible bag plant, because of the greater popularity, longevity, and production of gas. To install a fixed dome biogas plant, sufficient land is first required, preferably in the area surrounding the kitchen. The value of the land was not included in our cost estimation because: (1) households usually do not need to purchase the land for installing the biogas plant; and (2) after installation, the land can also be used for other purposes, because biogas plants usually do not produce a foul odour. In other words, there is no incremental cost associated with land for the biogas plant. It usually takes three to seven days to construct a biogas plant, and then another week to dry it out ready for use. The cost of masonry, labour, materials (sand, bricks, cement, pipe, etc.), and pipes, is a fixed cost of around PKR 50,282 (adjusted) (USD 502) [25]. In addition, a stove suitable for using biogas, LPG, or natural piped gas is required instead of a simple burner. This costs around USD 60 and has an expected life of 10 years [31] (we used the inflation adjusted price of PKR 7,201 (USD 72)).

In terms of operating costs, a 10m^3^ biogas digester needs around 10kg of wet dung per day mixed with an equal amount of water, in order to produce enough gas for an average family [32]. We did not include the expenditure on dung because usually biogas adopters have access to freely available animal dung [18]. However, labour hours are required to feed the plant and to collect the slurry (waste after using dung). We assume 45 minutes per day for these chores. Using the hedonic wage method [33], and the Pakistan minimum wage of PKR 500 per eight-hour day, this labour cost would be PKR 46.8. Hence, the annual labour cost per household is PKR 17,082 (USD 170). The stove also has a maintenance cost of PKR 332 (adjusted) (USD 3.3) per year per household [34].

#### Costs of LPG

This intervention requires a methane stove (as noted in the previous subsection), with a cost of PKR 7,201 (USD 72). It also requires a domestic LPG cylinder. The average cylinder costs around PKR 6,001 (price adjusted) with 10 years of life expectancy [31]. In terms of operating costs, the average consumption of LPG and unit value price were taken from the Pakistan Social and Living Measurement Survey 2014. The monthly mean consumption of LPG is around 6.35kg and the average price of the LPG is PKR 138.5/kg, meaning an annual cost of PKR 10,553 (USD 105) (weighted consumption, PSLM-2014). The stove also incurs a maintenance cost of PKR 332 (USD 3.3) per year per household (as noted in the previous subsection).

#### Costs of natural gas

This intervention requires a methane stove as well (as noted in subsection “Cost of biogas digester”), with a cost of PKR 7,201 (USD 72). It also requires piped gas connection, which involves connection charges of PKR 6000 (Sui Northern Gas, Pakistan) as well as PKR 8,844 for two days of labour for a gasfitter (Salary expert, Pakistan) and the cost of pipes. On average, 1.8 Million Metric British Thermal Units (MMBTU) (weighted consumption, PSLM-2014) of piped natural gas are consumed by each connected household monthly, and the average price of piped natural gas for the year 2014 was PKR 442, and the annual maintenance cost was PKR 332. In total, the operating cost for a household a year is PKR 9,921 (USD 99).

#### Costs of electric stoves

The cost of a medium-sized modern electric stove was around PKR 35,245 (adjusted) (USD 352) [21], and the life of an electric stove is usually around 10 years. Most households (almost 87 percent (PSLM-2014)) already have an electricity connection that is used for lighting. Therefore, we ignored the electricity connection cost in our analysis. In terms of operating costs, a medium size modern electric stove uses around 1500 watts per hour. A household usually cooks three times a day and spends 2–3 hours in the kitchen for cooking [35]. The price of per unit of electricity varies with the variation in total consumption, with higher consumption leading to a higher price per unit. We took the average electricity price of PKR 10.50/kwh, which is the amount paid by middle-class and lower middle-class households in Pakistan (using 101–300 kwh per month). The operating cost of an electric stove is therefore PKR 14,372 (USD 143) per year. In addition, there is an annual maintenance cost of PKR 332 (USD 3.3).

#### Costs of improved cooking stoves

As a fixed cost, the price of the ICS is PKR 1000 to 3000 [36] and it has a life expectancy of three years [17]. We took the midpoint price PKR 1,729 (USD 17) (adjusted for 2014) as a total fixed cost. In terms of operating costs, a household currently spends around PKR 3012 annually on solid fuel consumption (details are in the section “Fuel Savings”). An ICS can save up to 35 percent of fuel use [37]. Therefore, the fuel cost for the ICS is 65 percent of normal fuel cost, or PKR 1958 (USD 1 9.5) per year.

Similarly, each household spends between 30 minutes to 4 hours on average per day for biomass collection in developing countries [17]. We took the median time of 2 hours and 25 minutes per day per household for biomass collection. Using the minimum wage, a household bears PKR 42,375 (USD 423) as a labour cost annually, and total operating cost is PKR 44,333 (USD 443) annually.

#### Summary of costs

Table 2 presents a summary of the costs that a household bears annually and ten years of discounted operating costs that would be incurred by those households. The table shows that the biogas digester is the costliest intervention and LPG has the least cost, followed by natural gas. ICS has a reasonably low cost but its estimated useful life is only three years, as noted in the previous section.

**Table 2:** Summary of the costs for all interventions.

Interventions	For a HH (PKR)	10 years’ discounted cost for the entire country (million PKR) Approx.		
**Biogas**		DR 3%	DR 7.5%	DR 12%
Installation (FC)	50,282	3,203,571	2,830,599	2,552,137
Stove (FC)	7,201			
Labour cost (VC)	17,082			
Maintenance (VC)	332			
**LPG**				
Cylinder (FC)	6,001	1,656,526	1,423,392	1,249,334
Stove (FC)	7,201			
Fuel cost (VC)	10,553			
Maintenance (VC)	332			
**Natural Gas**				
Connection (FC)	6,000	1,656,590	1,445,002	1,287,030
Stove (FC)	7,201			
Labour cost (FC)	8,844			
Gas charges (VC)	9,547			
Maintenance (VC)	332			
**Electric Stove**				
Stove (FC)	35,245	2,502,715	2,187,786	1,952,659
Electricity charges (VC)	14,372			
Maintenance (VC)	332			
**ICS (discounted for 3 Years)**				
Stove (FC)	1,729	1,992,174	1,912,618	1,841,423
Fuel cost (VC)	1,958			
Labour (VC)	42,375			

Notes: HH = Households, FC = Fixed Cost, VC = Variable Cost, and DR = Discount Rate. Fuel cost, Labour cost, gas charges, electricity charges, and maintenance charges are on annual basis and estimated for a single household. The discounted cost is multiplied by the number of households that depend on solid fuels to estimate the total cost for all of Pakistan. 1 USD = 100 PKR (2014). Source: Authors’ calculations.

### Benefits

The benefits of the interventions include fuel saving, the cost averted due to illness associated with IAP, productivity gains, time saving, and environmental impacts. Some health impacts that are associated with IAP, but are difficult to measure, such as mental stress or psychological pressure, are not included in the analysis. Similarly, the environmental benefits at local and global level are not estimated because they are outside the scope of this study, as are changes in household consumption (other than through the direct costs of health care) or income effects on the pattern of consumption. As the benefits are generally similar across the five interventions, we summarise the benefits by type in the subsections below, rather than by intervention.

#### Fuel savings

The average consumption and prices of the biomass fuels such as firewood, agricultural residues, and animal dung were estimated from PSLM-2014 data. The average price of firewood is PKR 9/kg and average monthly consumption was 54kg per household. Similarly, crop residues and animal dung have prices of PKR 5.31/kg and PKR 4/kg respectively. Average monthly consumption of crop residues and animal dung were 29.57kg and 27.54kg per household respectively. Hence, the annual expenditure a household saves from avoiding the use of biomass fuels was PKR 5832 for firewood, PKR 1884 for crop residues, and PKR 1322 for animal dung. The expenditures on animal dung and crop residues can vary significantly, because households may collect these two fuels themselves and therefore not pay. We took the average of major biomass fuels’, which is PKR 3012 (USD 30.12), because usually households use a mixture of these fuels. Thus, a household that adopts cleaner fuels such as LPG, piped natural gas, biogas, and electricity can save PKR 3012 annually. This is the benefit for a household switching to biogas, LPG, natural gas, or electric stoves, where biomass fuels would no longer be required. In contrast, households that adopt ICS will keep consuming biomass. However, due to better efficiency they will save 35 percent of the total biomass cost [37], which is equal to PKR 1054 (10.5 USD).

#### Fertilizer savings

Biogas digesters have an additional benefit compared with all other interventions, which is the use of slurry (a semi-liquid mixture, typically of fine particles of manure or dung) as a fertilizer. The slurry can be used as an organic fertilizer for crops [25], and can save the household around PKR 600 (non-adjusted) in fertilizer costs per month; thus, annually it saves PKR 9,835 (USD 98) (adjusted) [38].

#### Health impacts

*Impact on mortality and morbidity*. We assume that universal adoption of any of the interventions would almost eliminate IAP-related mortality and morbidity for a household. According to the Pakistan Strategic Country Environmental Assessment by the World Bank, IAP accounts for 28,000 deaths per year in Pakistan. Around 1,376,000 disability adjusted life years (DALYs) are lost each year due to IAP, of which 82% is from mortality and 18% from morbidity [39]. In developed countries, about 1.5 percent of infant and child mortality is associated with IAP [18]. Therefore, we assumed 98.5% of mortality (27,580 out of 28,000) and morbidity (1,355,360 out of 1,376,000) can be averted by shifting entirely from solid fuels to clean fuels.

To estimate the value of statistical life (VSL), various models have been used in past. For example, Thaler and Rosen, (1976) [40] used a hedonic (quality adjusted) wage model, Cameron et al. (2010) [41] used contingent valuation methods, and Hutton *et al*. (2007) [17] used a human capital approach. Recently, Viscusi and Masterman (2017) [42] estimated the VSL for Pakistan by extrapolating from international studies and assuming an income elasticity of VSL equal to one. We converted the estimated VSL (0.248 million USD) into local currency and adjust it for inflation (PKR 21.45 million). Therefore, Pakistan can reap total benefits equal to PKR 591 billion (5.9 billion USD) by averting mortality due to IAP.

The second important health benefits arise from saving DALYs. As noted above, 18 percent of total DALYs lost is due to morbidity, which is 243,964 DALYs. We value DALYs using the human capital approach, based on the assumption that one DALY is valued at the average gross national income (GNI) of Pakistan for 2014, which is PKR 132,000 [20]. Hence in total, PKR 32 billion can be reaped as benefits by reducing IAP annually. Finally, the total benefits that Pakistan can gain is PKR 623 billion (6.23 billion USD) from reduced mortality and morbidity annually.

#### Health care cost savings

We assume that the people of Pakistan who get ill due to respiratory illness were taking medicine and visiting doctors during the time before their deaths. We make the simplifying assumption that one DALY lost equates to one year of illness. As mentioned above, 1,355,360 DALYs are lost due to IAP in Pakistan each year. The average length of stay in hospital for patients depends upon the level of severity [17]. We assume that 86 percent of these (1,165,609) are moderate cases, 12 percent (162,643) are severe cases, and 2 percent (27,107) are very severe cases. We further assume that moderate cases are not admitted to hospital, but visit hospital twice each year, severe cases are admitted to hospital for 3 days, and very severe cases are admitted to hospital for 5 days in each year [31].

The cost of a hospital admission is estimated at PKR 1071 per day. This includes medicine, radiology, labour, transport, patient’s attendees, food, and hospital fees [43]. The cost of visiting hospital (but not being admitted) is estimated at PKR 423, including the cost of medicine, transport, and hospital fees [43]. We used these costs after inflation adjustment (PKR 2465 per day for admissions and PKR 974 for visiting hospital).

In total, moderate cases cost PKR 2.22 billion per year, whereas severe and very severe cases cost PKR 1.20 billion and PKR 334 million respectively. So, in total the health care cost savings by eliminating IAP are PKR 3.81 billion (38 million USD). These benefits also occur equally for all of the interventions we are evaluating except ICS adoption, because the households that use ICS do not stop using solid fuels, and so will not eliminate IAP.

#### Productivity gains

We used the human capital approach and took per capita GNI to estimate the illness-free value of productivity gains. We took the same years lost (1,355,360) due to IAP from the previous subsection, and made similar assumptions. For example, out of total lost years, 86 percent are moderate cases who do not work for two days, 12 percent are severe cases who do not work for 3 days and 2 percent are very severe cases who do not work for 5 days in each year. In total this results in 2,954,685 lost days of productivity. The total value of this lost productivity is equal to PKR 1.08 billion (10.8 million USD). These benefits also occur equally for all five of the interventions we are evaluating except ICS.

#### Time savings

We estimated the two types of net time savings in our analysis. First, we estimated the time saved if households do not need to collect biomass fuels, and second, we estimated the time saved on cooking because of the use of more efficient stoves. We used per capita GNI to estimate the value of the total time savings [17]. The amount of time saved differs between the different interventions, depending on the amount of time savings they are associated with.

As stated earlier, an average household spends around 2 hours and 25 minutes per day for biomass collection. In the case of biogas plants, a household will need to spend almost 45 minutes per day feeding the biogas plant. By subtracting this time from the biomass collection time, a household that installs a biogas plant can get net time benefits of around 1 hour 40 minutes per day. Biogas also saves cooking time of around 42 minutes per day because of efficient cooking source (clean energy sources save utensils washing time and fire burning time) [44]. These 42 minutes can also be saved in case of LPG and natural gas interventions because of same stove attributes. Therefore, in total a household can save up to net 2 hours and 22 minutes in the case of biogas adoption. Usually, households spend 25 percent of saved time on income generating activities and the rest of the time on other social activities [44]. Thus, a household spends 35 minutes of their saved time on income generating activities and the wage of a minute is around PKR 0.764 according to GNI. Hence, if a household adopts biogas it saves PKR 26.7 daily, equating to PKR 9,759 (97.5 USD) annually.

Similarly, in the case of LPG adoption a household saves around 42 minutes due to efficient cooking and 2 hours 25 minutes by avoiding biomass collection. By taking 25 percent of time a household saves 47 minutes for income generating activities. In this way, time saving gives that household PKR 36 daily and PKR 13,104 (131 USD) annually. Likewise, the benefits from natural gas and electric stove are PKR 13,105 (131 USD) each. However, in case of ICS, wood collection time reduces by around 8 minutes because households require less biomass for cooking the same amount of food and cooking time saves around 14 minutes because of efficient cooking [45]. In total an ICS can save up to net 22 minutes per day and spends 25% on income generating activities, hence it saves an annual income of PKR 1,534 (15.3 USD) per household.

Moreover, we assumed 35 percent of benefits for ICS (except time and fuel saving benefits as they estimated separately) therefore, we also assume 35 percent reduction in exposure of IAP [19, 24]. However, ventilation conditions widely vary among ICS and due to this; these estimates may be considered as a poor approximation [17].

#### Summary of benefits

Table 3 presents a summary of the benefits for a single household per year and ten years of discounted estimated benefits (fuel savings, health impacts, health care cost savings, productivity gains, and time savings) for each intervention. It shows the potential benefits for those households that shift from solid fuel use to the clean fuels. LPG, natural gas, and electric stoves have similar benefits, while ICS has the least benefit because households do not stop using solid fuels.

**Table 3:** Summary of the Benefits for all interventions.

Interventions	For a HH (PKR)	10 years discounted benefits for the entire country (million PKR) Approx.		
**Biogas**		DR 3%	DR 7.5%	DR 12%
Fuel saving	3012	8,547,730	7,178,688	6,156,556
Fertilizer saving	9,835			
Mortality benefits	38,877			
Morbidity benefits	2,116			
Health care benefits	250			
Time saving	9,759			
Productivity gain	71			
**LPG**				
Fuel saving	3,012	7,679,991	6,449,930	5,531,562
Mortality benefits	38,877			
Morbidity benefits	2,116			
Health care benefits	250			
Time saving	13,105			
Productivity gain	71			
**Natural Gas**				
Fuel saving	3,012	7,679,991	6,449,930	5,531,562
Mortality benefits	38,877			
Morbidity benefits	2,116			
Health care benefits	250			
Time saving	13,105			
Productivity gain	71			
**Electric Stove**				
Fuel saving	3,012	7,679,991	6,449,930	5,531,562
Mortality benefits	38,877			
Morbidity benefits	2,116			
Health care benefits	250			
Time saving	13,105			
Productivity gain	71			
**ICS (discounted for 3 Years)**				
Fuel saving	1,054	755,962	725,369	697,991
Mortality benefits	13,607			
Morbidity benefits	741			
Health care benefits	88			
Time saving	1,534			
Productivity gain	25			

Notes: HH = Households. DR = Discount Rate, the 10 years discounted benefits are multiplied by the number of households that shift from solid fuel to the interventions to get the benefits for entire country. 1 USD = 100 PKR (2014). Source: Authors’ calculations.

## Results and discussion

Table 4 presents the BCRs and NPVs for the five interventions. All have BCRs above one except ICS, implying that all the IAP reducing interventions are beneficial except ICS. When households adopt ICS, they do not stop consuming solid fuels, and this could be the main reason for a BCR of less than one for ICS. Our estimated BCR for ICS supports the World Health Organization’s study [17] conducted at the regional level, where they found the BCR of ICS was less than one in all the regions of the world except one (EMR-B). Our estimates for ICS also support Harvard University’s randomized trail study Hanna et al., (2016) [22], which was conducted in India in 2016, in which they found ICS was not a beneficial intervention. On the other hand, our estimated BCR of ICS contradicts those of Aunan *et al*. (2013) [26], Isihak *et al*. (2012) [27] and García et al. (2010) [28], who all found BCRs of greater than one.

**Table 4:** BCRs and NPV for all interventions.

Interventions		Discount Rates		
		3%	7.5%	12%
Biogas	BCRs	2.67	2.54	2.41
	NPV (PKR)	293,579	188,140	126,037
LPG	BCRs	4.64	4.53	4.43
	NPV (PKR)	338,161	232,632	170,476
Natural Gas	BCRs	4.64	4.46	4.30
	NPV (PKR)	336,912	229,102	165,603
Electric Stove	BCRs	3.07	2.95	2.83
	NPV (PKR)	287,290	190,419	133,363
ICS	BCRs	0.38	0.38	0.38
	NPV (PKR)	Negative	Negative	Negative

Notes: Authors’ calculations, USD 1 = PKR100 (2014).

Universal adoption of LPG has the highest BCR in our analysis. LPG has special requirement for connection, and consequently a low initial cost. This is the main reason for its high BCR. Surprisingly, our estimated BCR for LPG contradicts Hutton *et al*. (2007) [17], but corroborates, Isihak *et al*. (2012) [27] and Malla *et al*. (2011) [16]. Similarly, the BCR for biogas digester adoption was greater than one, and our estimates support the previous studies of Abbas *et al*. (2017) [25] and Limmeechokchai and Chawana (2007) [30]. Biogas plants had the least positive BCR, perhaps due the high initial cost.

The second most beneficial alternative to solid fuel is piped natural gas (close to LPG), with a BCR of 4.64, 4.46, and 4.30 at 3%, 7.5%, and 12% discount rates respectively. Similarly, the BCR of electric stove adoption was found to be 2.95 (at the 7.5% discount rate). To our knowledge no previously published study has carried out cost-benefit analysis for piped natural gas and electric stoves. Although electricity is the cleanest alternative, the energy infrastructure in Pakistan is poorly managed and there are frequent power blackouts, so households do not currently rely on electric stoves. Thus, for electric stoves to be a feasible solution to IAP, these supply problems will first need to be addressed. The estimated BCR does not account for the costs related to this infrastructure, and so the BCR of electric stoves is likely overestimated. Similarly, piped natural gas is currently only available in urban areas in Pakistan [18]. To extend piped natural gas to rural households would require significant infrastructure investment, which is not included in our analysis, and thus the BCR for piped natural gas is likely to be substantially overestimated. This is why we rank LPG as the most beneficial intervention.

We did not calculate the internal rate of return or return on investment because of the considerably high BCRs. We estimated the NPV of each intervention as well, with NPV in Pakistani rupees evaluated at discount rates of 3%, 7.5%, and 12%. The results are consistent with the BCRs. The NPV of biogas, natural gas, LPG, and electric stoves are positive, while NPV is negative for ICS at all levels of the discount rate. The NPV also suggests that the most beneficial intervention is adoption of LPG, with natural gas adoption as the second most beneficial intervention (again close to LPG). However, as stated earlier, these results do not account for the substantial infrastructure investment that would be required to extend piped natural gas to rural areas of Pakistan.

Unfortunately, pollution control strategies have to be carried out in the presence of uncertainties. The willingness to pay for clean energy sources e.g. LPG, piped natural gas, biogas, and electric stoves varies because of socio-economic and geographic factors. For instance, households that have higher income and education might be willing to pay more for cleaner cooking fuels. Likewise, households living near forested areas may have lower willingness to pay for alternatives due to the easily available of firewood for cooking and heating purposes. Similarly, households living in hilly areas, where finding alternative energy sources is difficult and provision of piped gas is expensive, might have higher willingness to pay for cleaner fuels. In a similar way, the estimation of benefits can also be uncertain. For example, while estimating the VSL there are two major uncertainties such as; first, assuming 98.2% aversion may be too high for developing countries. Even when households shift from biomass consumption to cleaner fuels, they still have a chance of getting pollution related diseases (e.g. respiratory infections) because of the outdoor air pollution. Hence, the benefits can be lower than we have estimated. Therefore, considering these uncertainties we undertook sensitivity analysis and present the results in Table 5.

**Table 5:** BCRs for pessimistic and optimistic scenarios.

Interventions	Scenario	
	Pessimistic	Optimistic
**At 5 Percent Fluctuation (discount rate 7.5%)**		
Biogas	2.29	2.80
LPG	4.10	5.01
Natural gas	4.04	4.93
Electric stove	2.67	3.26
ICS	0.34	0.42
**At 10 Percent Fluctuation (discount rate 7.5%)**		
Biogas	2.07	3.10
LPG	3.71	5.54
Natural gas	3.65	5.46
Electric stove	2.41	3.60
ICS	0.31	0.46

Authors’ calculations.

We looked at pessimistic and optimistic scenarios for the uncertainty analysis. In the pessimistic scenarios, we added five (or ten) percent to costs and deducted five (or ten) percent of the benefits at the discount rate of 7.5%. Similarly, in the optimistic scenarios we subtracted five (or ten) percent of the costs and added five (or ten) percent to the benefits at the discount rate of 7.5%. The results show that, even in the most pessimistic scenario, the BCRs of the interventions are above one, except for ICS.

## Conclusions

Owing to IAP, almost four million people annually die prematurely and millions more face serious diseases. The adverse health impacts place a great burden on national health budgets, increase medical expenditures, and reduce the overall productivity of the economy. With the financial loss, the welfare of those adversely affected by IAP and their families deteriorates, yet more than three billion people depend upon solid fuel consumption, even though it is the major contributor to IAP. Many local, international, government, and non-government organizations have intervened to control IAP by subsidising and investing in cleaner fuel adoption. However, previous cost-benefit studies have focused on LPG, biogas, and ICS, and few studies have considered the benefits and costs for a single country. We extended earlier analyses by including consideration of piped natural gas and electric stoves, alongside adoption of LPG, biogas, and ICS.

We followed the guidelines of the World Health Organization (WHO) to conduct cost-benefit analysis in Pakistan. Data from various sources such as PSLM, World Bank, WHO, UN, SNGPL, and earlier published studies were used to conduct the analysis. All interventions (except ICS) were forecasted for the period of 10 years with discount rates of 3%, 7.5%, and 12%. Because of the numerous assumptions in the analysis, we also undertook a sensitivity analysis by varying the extent of benefits and costs, and the results of the sensitivity analysis show that our main results are relatively robust.

It is challenging to rank the interventions because of different scales of the interventions, timing, and risk factors. However, based on our analysis, we conclude that LPG adoption is the most beneficial alternative. It has the highest BCR and NPV perhaps because it has the lowest adoption cost. Piped natural gas and electric stoves ranked second and third respectively. However, electric stoves and piped natural gas would require significant infrastructure investment in Pakistan, which is not accounted for in our analysis. Nevertheless, other developing countries that do not face high infrastructure costs to adopt piped natural gas and electricity may find them to be more cost-effective alternatives. Although the adoption of biogas plants ranked at number four because of the highest installation cost, it can be highly useful in rural areas where other clean energy sources (natural gas, LPG, and electricity) are not available. Finally, we conclude that the use of ICS to reduce IAP is not beneficial as the BCR for ICS was less than one and NPV was negative. The health gains from ICS are the lowest because households do not stop consuming solid fuels when using ICS.

We faced several challenges in monetizing the benefits and costs due to non-availability of credible data. Arguably, several of our assumptions were very close to the real life; however, we also accounted for uncertainty with sensitivity analysis. Even in the most pessimistic scenario, the BCRs of the alternative interventions (clean fuels) were greater than one, implying that the interventions are beneficial. Our findings can be used to guide governments and other stakeholders in choosing the most beneficial intervention to reduce IAP.

## Supporting information

S1 Table Data sources. (DOCX)

S2 Table Data. (XLSX)
