# Supplementary material for: Interventions to mitigate indoor air pollution: A cost-benefit analysis
Source: PLoS One. 2021 Sep 24;16(9):e0257543. doi: 10.1371/journal.pone.0257543 (PMC8462720; doi:10.1371/journal.pone.0257543)
Supplement: S1 Table — (DOCX) [file pone.0257543.s001.docx]

**S1 Table. Data sources.**

| **Variable** | **Sources** |
| --- | --- |
| Prices of LPG, Firewood, Natural gas | PSLM-2013-14 |
| Price of electricity | Ministry of Water and Supply |
| Total population | World Bank data |
| Average household size | World Bank data |
| Fixed cost of Biogas plant, Operating cost of Biogas plant, price of slurry | Bio Energy Technology Application Pakistan,  published literature |
| Minimum wage rate | Pay Check Pakistan  https://paycheck.pk/main/salary/minimum-wages/minimum-wage-in-pakistan-2014 |
| Fixed cost of natural gas connection and plumber salary | Sui Northern Gas Pipelines limited (SNGPL)  https://www.sngpl.com.pk/web/  <https://www.salaryexpert.com/salary/job/plumber/pakistan> |
| Population of children | United Nation databank |
| Mortalities and morbidities (DALYS) | World Health Organization,  published studies |
| Per capita income | World Bank data |
| Regional population, population of  working age | Pakistan Bureau of Statistics |
| Number of days spent in bed due to illness, fuel collection time, time spent on economic activity, costs of health care, operating cost of LPG, natural gas, electricity, and fixed cost of LPG, natural gas, and electric stoves. | PSLM-2013-14  and  published literature |
